# A novel approach for determining instantaneous centers of rotation of the mandible with an intraoral scanner: A preliminary study

**DOI:** 10.1371/journal.pone.0285162

**Published:** 2023-05-03

**Authors:** Arpad Safrany-Fark, Balazs Laczi, Antal Nagy, Laura Lengyel, Jozsef Piffko, Emil Segatto

**Affiliations:** 1 Department of Oral and Maxillofacial Surgery, Albert Szent-Györgyi Medical School, University of Szeged, Szeged, Hungary; 2 Faculty of Science and Informatics, Department of Image Processing and Computer Graphics, Institute of Informatics, University of Szeged, Szeged, Hungary; Medical University of South Carolina, UNITED STATES

## Abstract

**Objectives:**

Recording and reproducing mandibular movements have been of key importance in the practice of dentistry for over a century. Recently, it has become possible to use digital technologies for these tasks. This study presents a preliminary method to try to identify the mandibular instantaneous centres of rotation based solely on intraoral scanners.

**Methods:**

The dentitions of four participants were scanned, multiple inter-occlusal registrations and buccal scans were performed in closed and opened positions. Blender software was used to align the meshes during the post-scan digital workflow. Bite alignment accuracy was assessed and then improved with a strict exclusion protocol. An automated algorithm was used to find rotations between closed stage and open stage meshes.

**Results:**

Our exclusion protocol reduced the bite alignment error significantly (p = 0.001) and the root-mean-square error value of the meshes decreased from 0.09 mm (SD = 0.15) to 0.03 mm (SD = 0.017). However, the remaining translational error caused an unexpectedly large shift in the axis of rotation (mean = 1.35 mm, SD = 0.77) with a 41.83: 1 ratio. As found in other studies, our results showed even a small amount of error during registration can shift the axis of rotation a large amount. This phenomenon will compromise the results of common pantographic methods which assume a rotation axis of the condyle. It also adds valuable information to the concept of instantaneous centers of rotation by revealing their true characteristics.

## 1 Introduction

Recording and reproducing mandibular movements have been important in the practice of dentistry for over a century [1]. According to the traditional description of complex motional characteristics of the lower jaw, the first part of the opening phase may consist of only rotational movement [2,3]. However, the existence of a true (terminal) hinge axis (THA) has been debated [4,5]. The movements of the mandible have traditionally been recorded and reproduced with analog instruments, but recently digital methods have been developed to modernize the instrumentation [6]. Some of these evolved from the traditional face bow registration techniques and the use of anatomical information gained from three-dimensional radiological imaging [7–9]. Other techniques use digital pantographs or optoelectronic motion registers to record patient-specific dynamic information [10–12].

This study presents a method for patient-specific registration of axes of rotation based solely on intraoral scanners. A patient-specific input for digital articulation from the same device that is used to scan the dental arches would be optimal. However, the complexity of jaw motion makes the development of such instruments challenging. The current paper focuses only on the initial stages of such a development process as a preliminary study of a method. The limited clinical applicability of the methodology at this stage must be considered, as it might need further significant changes and improvements; however, it may act as the basis for a development process of virtual articulators based on patient-specific clinically obtained information rather than predefined anatomical norms. Our main objective was to develop the post-clinical stages of data analysis relatively unaffected by factors that could be improved in later stages of our work (e.g., different strategies of inter-occlusal recording and improvements in scanner accuracy).

The technical and scientific superiority of more sophisticated methods, such as ultrasonic, optical, electromagnetic, and magnetic jaw-tracking systems, as well as real-time magnetic resonance imaging and optical marker-enhanced cone-beam computed tomography, is unquestionable. These devices are the prime tools for dynamic functional analysis of mandibular movement [13–15]. However, a rapid, radiation-free technique with lower equipment demands and acquisition costs might have a relevant place in the instrumentation for both clinical and research use as long as its limitations are respected.

### 1.1 Theoretical background of our method

Intraoral scanners generate digital models of the dental arches and an inter-occlusal record captured by buccal scans, and these models can be exported in.stl files [16]. These files contain three-dimensional representations of the surfaces of the teeth and surrounding tissues in a triangle mesh [17]. The buccal scans record the spatial relationship of the upper and lower arches. This information is then used to align the meshes of the upper and lower arches, which were scanned separately.

After scanning the upper and lower arches, the scans can be duplicated multiple times before the buccal scanning phase. These duplicates can be reopened separately, and the buccal scan and alignment process can be repeated in different mandibular positions. Theoretically, if the first buccal scan is performed in a closed position and the second buccal scan is performed in a more open stage during closure, the result is two pairs of identical meshes with the sole difference being their location in virtual space; thus, the vertices of the meshes can be handled similarly in a 3D environment as marker points are used in 2D investigations of the instantaneous center of rotation (ICR) of human joints [18]. The ICR theory of the temporomandibular joint (TMJ), introduced by Grant, challenged the THA concept [19]. The ICR describes the position of the center of rotation at any instant during simultaneous rotatory and translational movement of the mandible. The ICR describes the position about which an object seems to be rotating at a given instant [20,21]. Such motion differs from THA because the center of rotation shifts along a path. The original graphic method of Reuleaux’s used by Grant to determine ICR was reformulated many times in the past decade [18–20,22,23]. Renewed 3D successors of 2D methods based on the two-position theory of kinematics were also reported in the literature [24]. Although the ICR is becoming more prevalent for modern explanations of mandibular movement, some authors have suggested that the use of the ICR position in the determination of the rotation axis for clinical dental procedures has its limitations [21].

## 2 Materials and methods

Four members of our medical staff served as participants in the study. It is important to state, that our aim at this stage was not to test our methodology in a relevant clinical situation, but to reduce every detrimental factor and test the method relatively free of clinical and technological errors, such as patient cooperation and scanner failure.

This study was approved by the Human Investigation Review Board of Szeged University (approval no.: 43/2020-SZTE). All participants were previously educated about the study concept and participated voluntarily, and written informed consent was obtained. Upper and lower scans were obtained with 3Shape TRIOS (Copenhagen, Denmark) (Software build 1.7.9.1) by the same operator, and the postprocess was carried out before buccal scans were made. After the postprocess, the project was duplicated 17 times (18 scans for each of the four participants).

During our intraoral scanner-based registration, we calculated the axes from the positional differences between a mesh pair for the lower dental arches. One mesh was positioned in an open stage in the course of closure; the other was in a closed state after the first contact. Our algorithm calculated the most accurate axis of rotation that could transfer the open mesh to an overlapping position with the closed mesh. However, our registration method produced an axis whether or not any translational component (or any type of error) was present beside the rotational movement. According to the ICR theory, these translational components occur naturally; however, artificial errors during inter-occlusal registration or the alignment process can also affect the final mesh position. Thus, our method produced ICRs technically; i.e., an axis about which our mesh seemed to be rotating.

### 2.1 Inter-occlusal record

**Fig 1:**
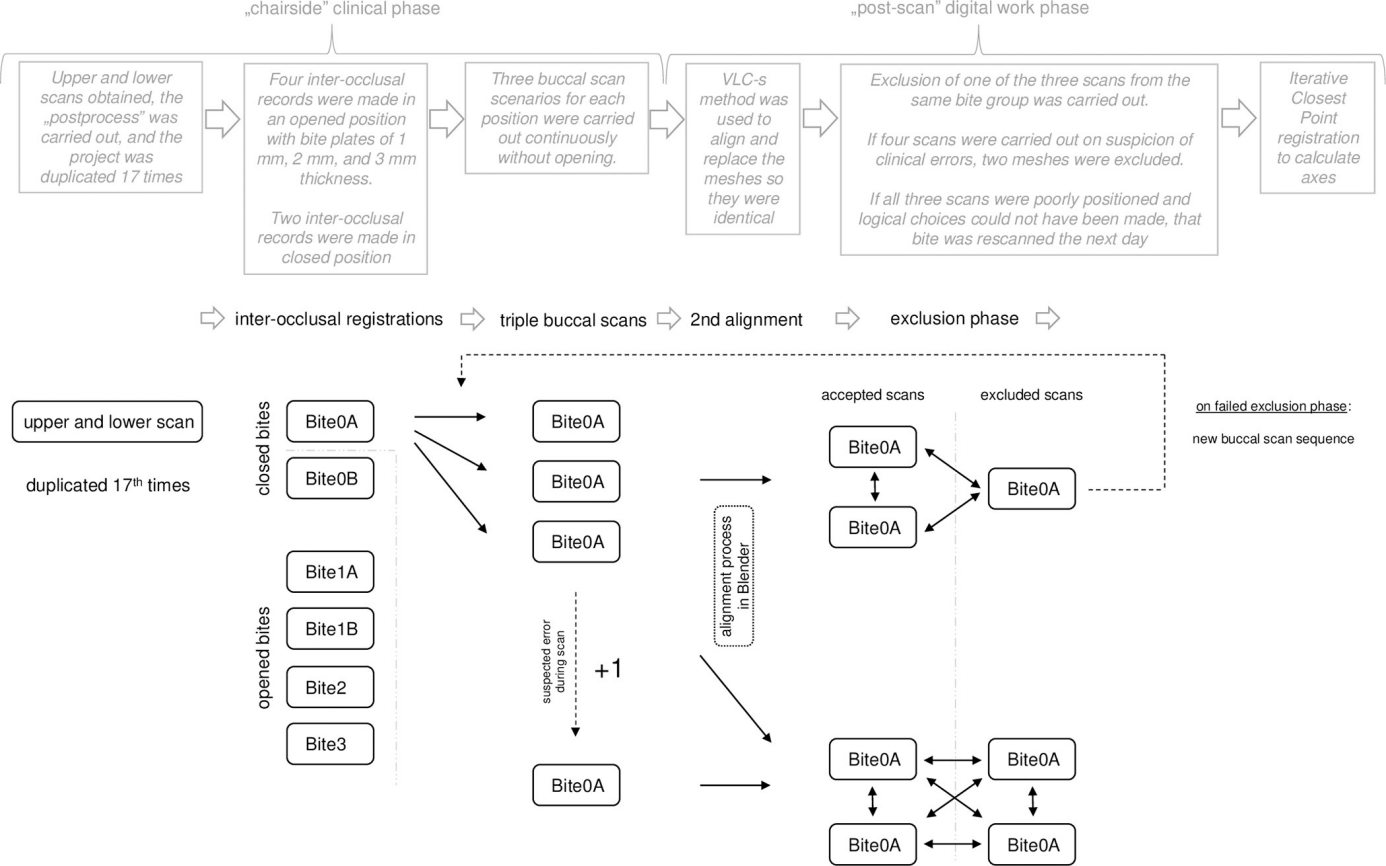
Schematic diagram of the workflow. Schematic figure of the registration process during the different clinical and computational phases.

Six inter-occlusal registrations were made: two in closed and four in opened positions for each participant. Each position was scanned three times continuously without opening to control bite alignment accuracy separately from clinical procedures (Fig 1, S1 Text). Preliminary investigations showed that the alignment accuracy was highly unstable, which is consistent with the findings of Nilisson et al. [25]. On average, two of three bite scans were placed relatively close to each other, while one of the three scans was positioned relatively further away. We eventually decided to carry out three scans continuously without any movement, but only the best visually-selected two scans of three were used for subsequent processing. These consecutive buccal scans that were carried out in the same inter-occlusal record formed *a group*; for a more fluid formulation, we will henceforth refer to them using the colloquial term *"bite groups"*.

Due to the stationary position that had to be maintained for a long period of time, we used a new composite-based method instead of the traditional methods of inter-occlusal registration (S1 Text).

Four inter-occlusal records were made in an open stage during mandibular closure. Plates of 1 mm, 2 mm, and 3 mm thickness were formed from dental composite materials and subsequently light cured before use to maintain vertical dimension. These bite groups are referred to as Bite1A, Bite1B, Bite2, and Bite3; see Fig 1.

Two “closed” inter-occlusal registrations were also made at the first contact (Bite0A and Bite0B) with flowable composite material applied on the palatal cusps of the upper first molars and premolars.

If any error was detected during the buccal scan, it was repeated immediately (such a repeated scan was needed because of increased saliva flow that was depicted on the buccal scan image; that bite group consisted of four scans instead of the normal three, two of which were excluded later; see Fig 1).

As a separate control tool for our methodology and the scanner’s in built bite alignment accuracy, we also performed scans on a pair of casts mounted in an articulator (SAM, München) in an open position and in a closed position three consecutive times without any movement of the articulator to exclude any clinical error caused by saliva, patient movement, or other chairside factors. We compared these results with our clinical results.

### 2.2 Post-scan digital workflow

The basic idea behind our study is that, if upper scans of all 18 registrations are identical, then they are presumed to be positioned identically in the 3D space. Nevertheless, if the lower *(also identical)* scans kept their unique positions as determined by the buccal scans, then the individual ICRs of the opened lower meshes (collated to the closed lower ones) could be calculated.

However, two obstacles prolonged the digital workflow:

- The 3shape software positioned the upper mashes randomly in digital space, but not in identical positions, which made another, time-consuming alignment process inevitable.
- The other unfortunate trait of the scanner, was that although the in-built post process feature had been carried out before duplication, it is mandatory to repeat it after the buccal scans. This event recalculated and altered the previously prepared surfaces of the upper and lower scans as well, thus the duplicated meshes were no longer identical.

**Fig 2:**
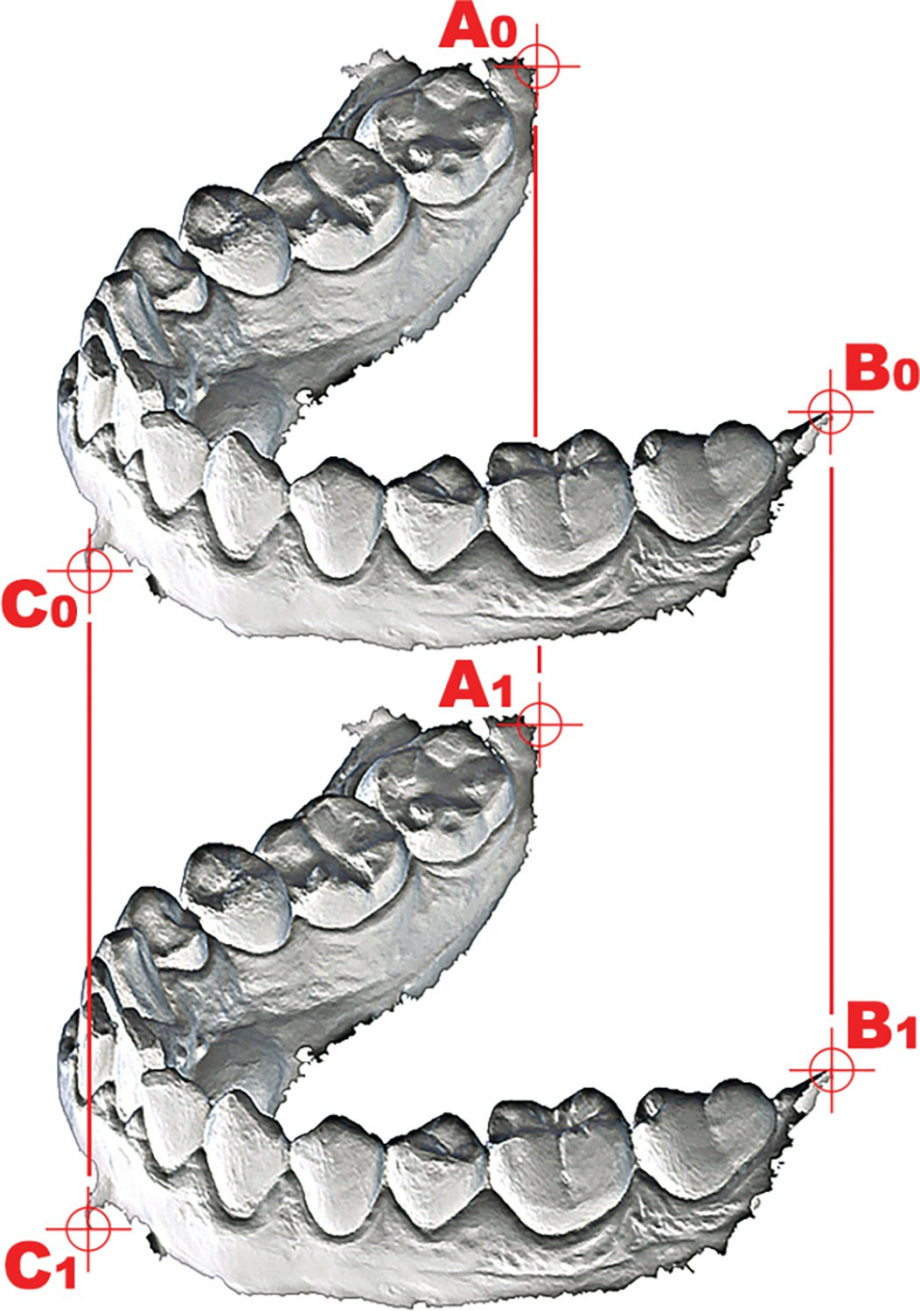
Schematic figure of alignment by CLVs.

These circumstances prolonged the post-scan digital work significantly and made it difficult to conduct large scale studies. It reduced the verifiability of the scanner and obstructed monitoring of the alignment accuracy, which would have been desirable [25]. To eliminate these problems, we used an alignment process combined with a substitutional phase carried out in Blender 2.79.2 (Blender Foundation, Amsterdam), which is an open-source general-purpose modeling program. The developed modeling method uses characteristically located vertices (CLVs) along the gingival edges of meshes. These CLVs stayed relatively stable after the postprocess, and can be located on all altered mesh surfaces. All upper and lower scans were aligned and substituted by these CLVs until they were in their correct position, and they were identical point sets (Fig 2). For detailed description of the CLVs method, see S1 Video and S2 Text.

### 2.3 Algorithm description

**Fig 3:**
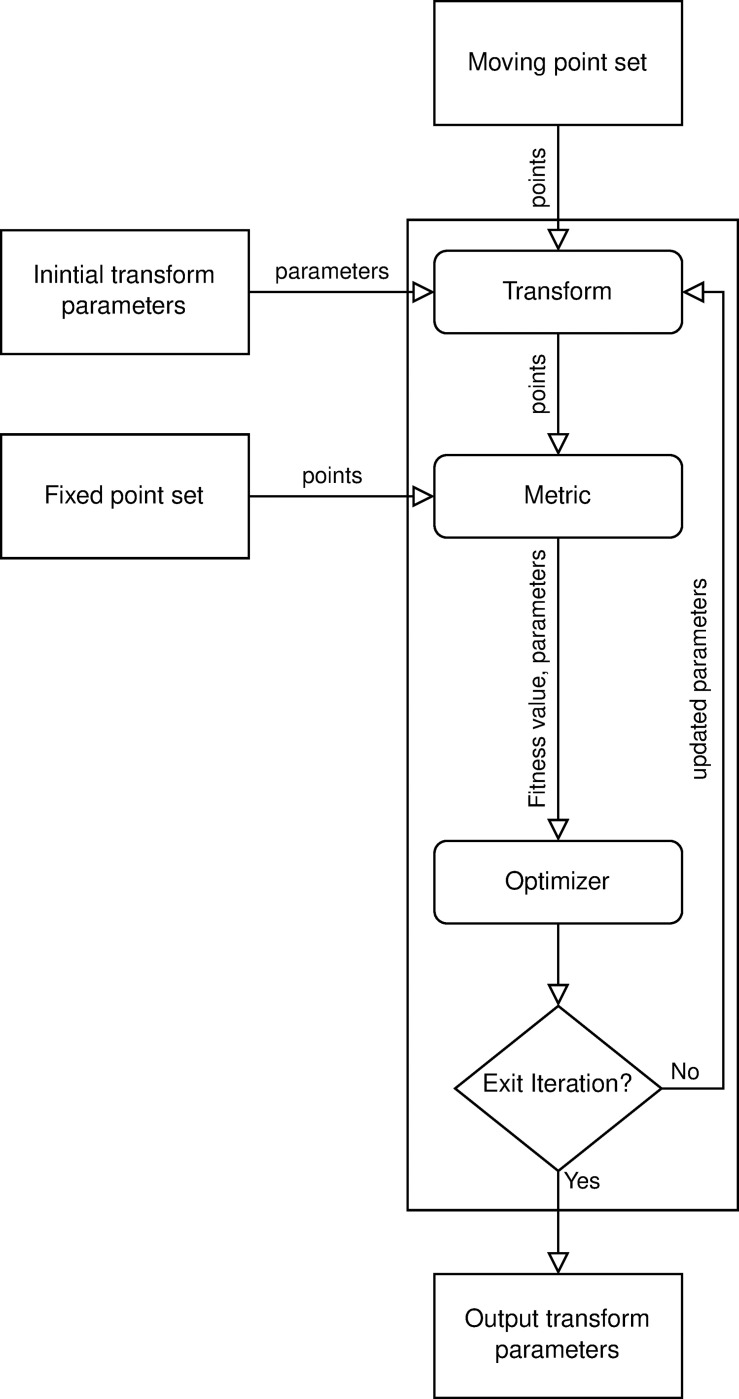
Schematic figure of the Iterative Closest Point registration method. In every iteration the optimizer was fine tuning the transformation parameters to find the best fit between the fixed and moving point sets. The moving point set was always the target of the transformation and the metric was designed to measure the fitness after the transformation. The iteration stopped if the optimizer could not find a better solution or if it reached a maximum number of iterations.

Registration methods can be used to find a predefined type of transformation between two images. In our work, instead of standard images, 3D surfaces were the targets of these registrations. These meshes can be handled as point sets. The positions of the points in 3D space are the relevant information in our case, not standard image properties such as color intensity. The previously manually fitted identical 3D scanned surfaces were the inputs of our automatic refining method. Every point in the moving point set had a pair in the fixed-point set, thus the input 3D surfaces could be handled as simple point sets. We used the Iterative Closest Point algorithm [26] with a simplified Euclidean distance metric, and Levenberg Marquart Optimizer (Fig 3) [27,28]. We implemented a special rotation transformation about an arbitrary axis according to Rodrigues’ rotation formula [29]. This transformation was defined by a point on the arbitrary axis with three parameters; i.e., two angles defined the direction of the axis and one rotation angle around the given axis.

We validated the proposed algorithm on 24 artificially transformed but clinically gained meshes, with the transformations carried out in Blender software. We calculated the closest point on the resulting axis and the nearest centroid point of the fixed-point set. This point was the center of a 20 cm length axis that was used for visualization and measuring the accuracy of our algorithm. The algorithm determined the endpoints of the rotation axis with 0.043 mm of mean error (SD = 0.053) and the artificially performed rotation had 0.0000329 degrees of error (SD = 0.0000295) on average. To describe the positional differences of two meshes, the root-mean-square error (rms_error) was calculated based on the distance of each point pair. The remaining rms_error after the transformation was 0.000585 mm (SD = 0.000704).

### 2.4 Graphical representation and evaluation of the axes

#### 2.4.1 Graphical representation

The calculated axes were graphically represented in Blender for further visual inspection by two points that were 200 mm apart (the *start point* and the *end point* of the calculated axes).

After the phase of exclusion, all four cases consisted of 12 scans. Four were in a closed and eight were in an opened position. This resulted in 32 axes per case that could be divided into four sets of axes, each consisting of eight axes that belonged to the same closed scan. We found that the position of the axes usually followed the same pattern in different sets. Thus the positional differences of the closed meshes caused an “en-masse” transition of the axes. However, this transition always slightly altered the relative position of the axes to each other.

The first set of axes for all four participants were represented separately in 2D figures on the level of the midsagittal plane as the axes penetrated the plane. These figures had a solely demonstrative role of providing better insight on the topic.

We also present the most centrally located axis within all four sets to demonstrate the relative position of the sets to each other.

#### 2.4.2 Analysis and evaluation

The main characteristics that can describe the positional differences of two axes are their distance and angular deviation. These can describe the three-dimensional displacement of the axis of rotation that occurs after the “displacement” of one of the scans that was used for calculating the axis.

As an example, we took three meshes of the lower arch, one for each of the following bites: Bite0A, Bite1A, and Bite1B. We calculate the axis of rotation for Bite1A and Bite1B “opened” meshes rotated on the same “closed” mesh of Bite0A. The differences in the positions of the two resulting axes are caused solely by the positional differences of the Bite1A and Bite1B meshes.

To keep the analyzed data within a reasonable limit, we decided that further evaluation on interaxial differences should be calculated only within the same bite groups at this stage. This provided information on the effect of bite alignment inaccuracy relatively free of clinical error. The distances of the axes were calculated on the *start points* and *end points* of the axes, and the means of these values were used as the distance/displacement of the axes of rotation (d_axis_), similarly to Mehl’s study [30]. As we described the distance of two meshes by their rms_error value, we can also describe the effect of this rms_error on the displacement of the axis of rotation by calculating the root-mean-square error caused by the rotation axis displacement ratio or “*error caused displacement ratio*” (d_axis_/rms_error = EcD_ratio). The angular deviation of the axes was also calculated. This also provided an absolute value of the deviation, which might be suitable for statistical analysis; however, all information about the orientation is lost, which highly limits the use of these results.

Statistical analysis was performed using IBM SPSS Statistics (Chicago, IL, USA) and Microsoft Excel (Redmond, WA, USA) software. SigmaPlot (San Jose, CA, USA) was used to graph the data.

## 3 Results

### 3.1 Statistical evaluation of exclusion

To justify the exclusion of one mesh, the rms_error values of all meshes within the same bite group were analyzed. We compared the rms_error values between the spared meshes (N = 24) to the values between the spared and excluded ones (N = 54). To prove the significant difference between the groups, we used Student’s *t*-test. The null hypothesis was that the average mean difference was zero and we used 0.05 as the alpha value. The calculated *t*-value was −3.418 and the calculated p-value was 0.001. Because the absolute value of the *t*-value was bigger than the critical *t*-value (2.000 according to the Student’s *t* distribution table, using the two-tailed version with 0.05 alpha, 95% confidence level and 60 degree of freedom) and the p-value was less than the alpha value, the null hypothesis failed, so there was a significant difference between the excluded and the spare ones. We also found similar results in the case of each participant separately (Case I.: p = 0.004; Case II.: p = 0.0003; Case III.: p = 0.003; and Case IV.: p = 0.031).

The mean of the rms_error between all included and excluded meshes within the groups was 0.09 mm (SD = 0.15). However, this value reduced 0.03 mm (SD = 0.017) within the included group, while the excluded ones compared to the spared ones had a 0.12 mm (SD = 0.177) mean rms_error. This shows that the excluded meshes were indeed placed significantly further from the spared ones. A separate, but still interesting, finding was that the control scans on the articulated casts provided a 0.03 mm (SD = 0.0068) mean error, which was similar to our results after exclusion. This might suggest that clinical factors, such as patient cooperation and saliva, might have a significant role in the misalignment of the excluded cases. Operator failure also might be a possible contributing factor; however, similar results from an independent source suggested that the cause of these failures are present universally [25]. Unfortunately, the built in features of the used scanner are probably the main reasons why large scale studies are hard to carry out to investigate and exclude these sources of error.

### 3.2 Graphical representation

**Fig 4:**
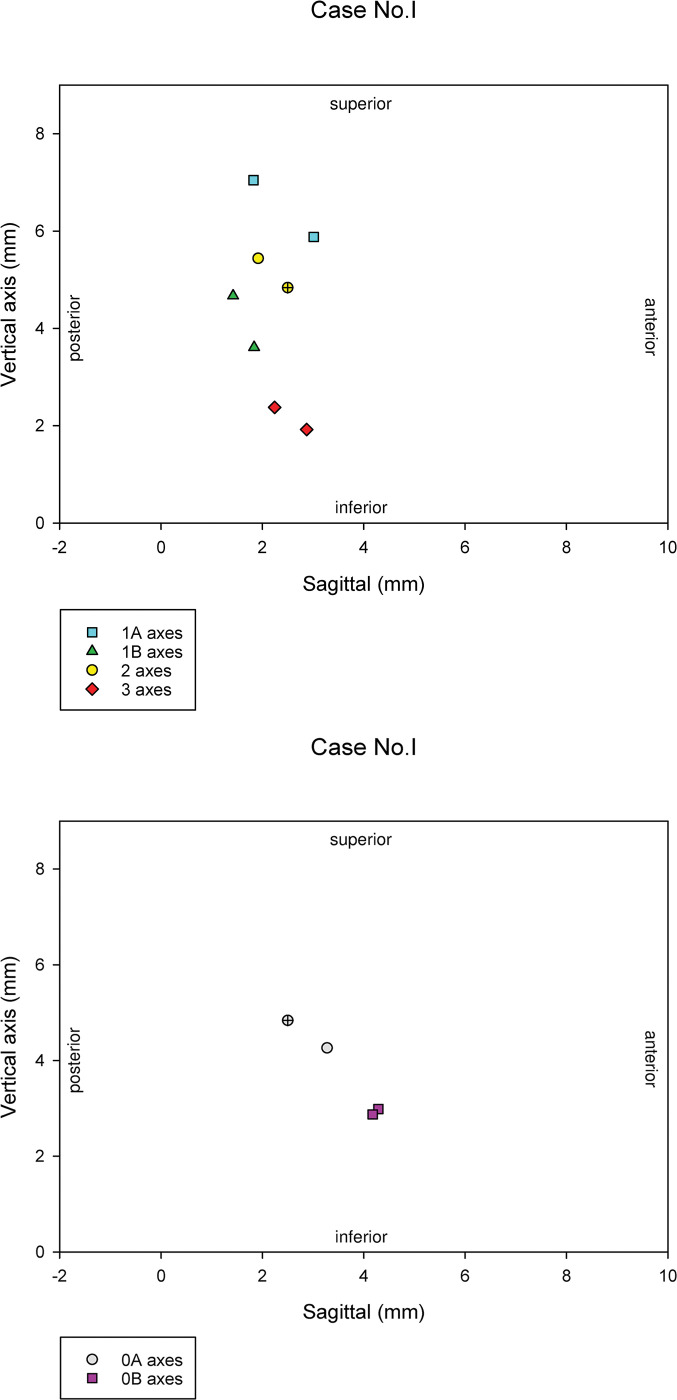
Graphical representation of the axes. The first set of axes for Case I. in 2D, on the level of the midsagittal plane, as the axes penetrate the plane. Fig 4A show the axes calculated as the first nonexcluded scan of the Bite0A position was used as the target of rotation for all nonexcluded scans of all opened bites. The opened scan of most centrally positioned axis (“x/+” marked symbols on the figures) was used to show the effect of error on the closed bites, thus all nonexcluded closed scans were used as the target of that chosen opened scan, shown on Fig 4B.

For further inspection, we show the first set of axes (Figs 4A, 4B and S1A–S1H.) with the relevant variables describing the differences in the axes of the same bite groups (S1 Table). The axes of the same bite groups are located relatively closer, thus inter-occlusal registration greatly affected the transition of the ICRs. Different types of inter-occlusal registration protocols may reduce this shift; however, large scale studies are needed to optimize the chairside protocol. The ICRs are distributed within an 8 X 8 mm area; but a clear pattern of the ICR shift is hard to describe due to the limited number of cases.

### 3.3 Further analysis and evaluation

**Fig 5:**
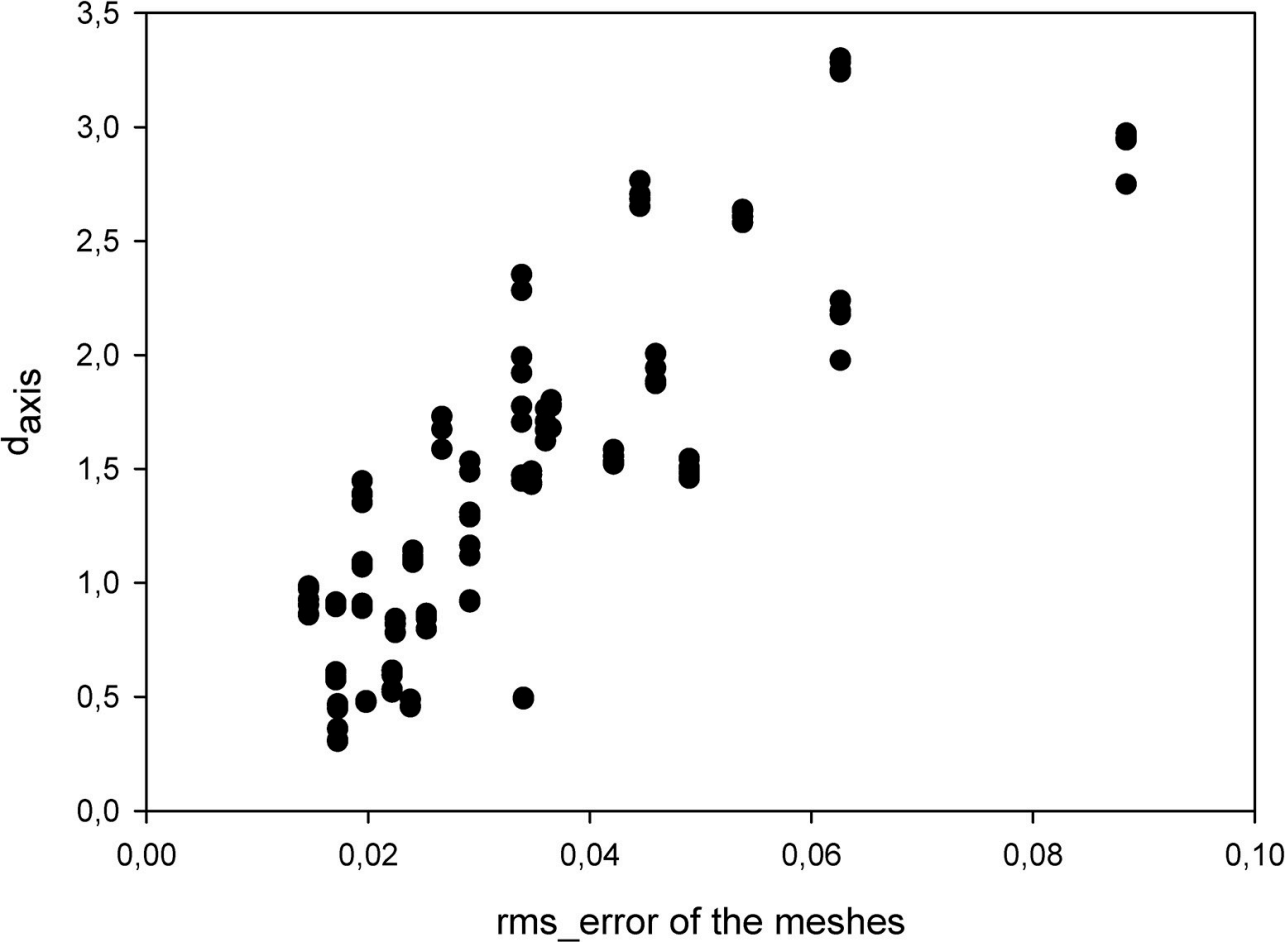
The effect of the rms_error of the meshes on the d_axis_ shows the EcD_ratio and its distribution.

The descriptive statistics showed that the mean value of the d_axis_ was 1.35 mm (SD = 0.77) with a mean 0.44° (SD = 0.29) of angular deviation between the compared axes. This high amount of displacement and angular deviation of the axes was caused by the previously described 0.03 mm (SD = 0.017) mean rms_error between the meshes, which might be a surprising result at first sight (Fig 5). Thus, we calculated the EcD_ratios for each pair of axes and found that the mean value of these EcD_ratios was 41.83:1 (SD = 14.89). This result showed that, within our methodology, a certain amount of displacement error of the meshes caused a displacement 40 times more of the calculated ICRs. The importance of this phenomenon cannot be exaggerated. Theoretically, in a classical metal marker and two radiograph based ICR calculation a 0.5 mm wide pen line used for Reuleaux’s graphical method would risk a 20 mm displacement of the calculated ICR solely caused by artificial error. However, this problem is still present as any digitalization process of biological information is a similar possible source of error (e.g. segmentation of CBCT or MRI images).

**Fig 6:**
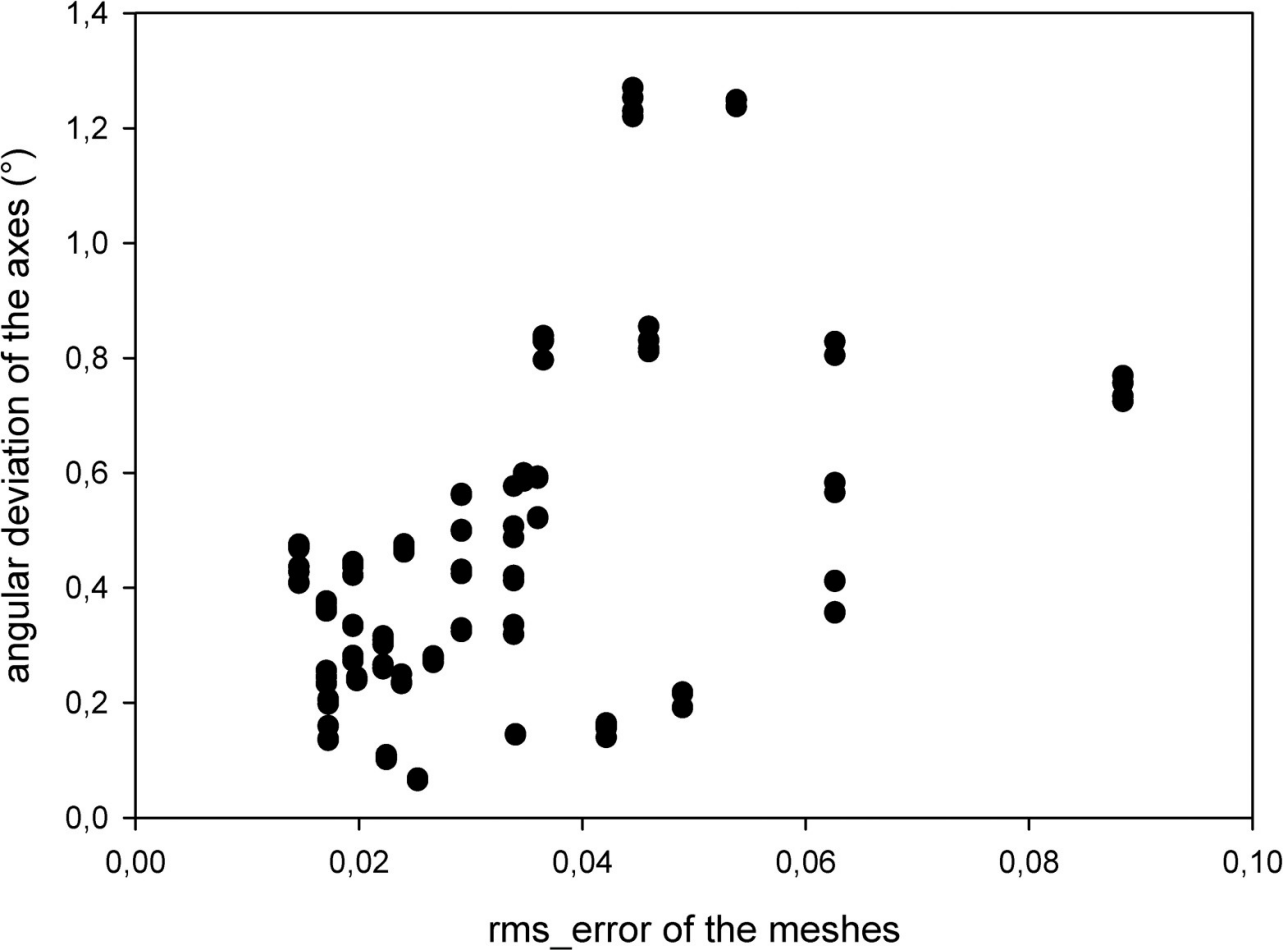
The effect of the rms_error of the meshes on the angular deviation of the axes.

We obtained a correlation value of 0.56486042 (p = 0.00000000) between the rms_error of the meshes and the d_axis_, and a value of 0.38325365 (p = 0.0000018) between the rms_error of the meshes and angular deviation of the axes (Figs 5 and 6). Angular deviation of the axes suggests that using 2D images might have limitations in ICR studies. Even if the sagittal plane that is used for the calculations was corrected to be perpendicular to an axis of a given instant, angular deviation might compromise the perpendicularity of the chosen plane to other ICRs.

**Fig 7:**
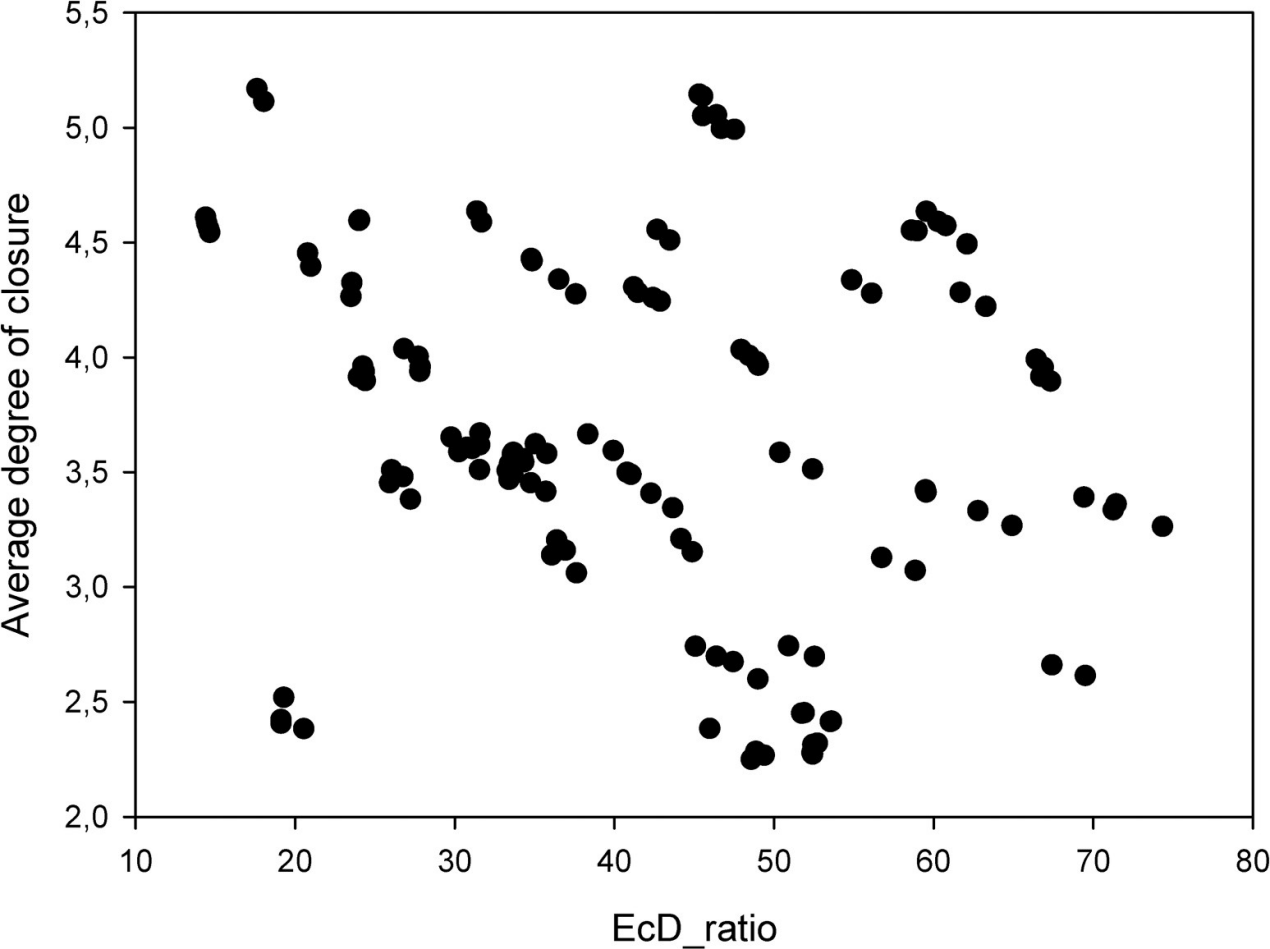
The amount of closure in degrees compared to the EcD_ratio.

We found a negative correlation tendency (−0.65903932, p = 0.00000000) between the amount of closure (hinge movement in degree) and the EcD_ratio (Fig 7). This negative correlation tendency suggests, that some real-time motion registering techniques might be more vulnerable to the translational error caused ICR displacement with higher frames per second, thus their EcD_ratio increases as the amount of rotation between frames decreases.

## 4 Discussion

Our results showed that our exclusion protocol significantly reduced the alignment error. Our preexclusion alignment accuracy was similar to the findings of Nilsson et al. [25], which suggests that alignment precision might be the weakest element and most limiting factor of current dental scanner technology. An accuracy of 0.09 mm of bite alignment may have a significant impact on clinical work. Even 0.03 mm is a concerning amount of error in some fields of dentistry. However, the rapid improvement of digital dentistry might eliminate this obstacle soon and reduce the alignment error which caused a shift of the axes to a certain level that might be tolerable for further assessment and improvement of our clinical methodology. Focusing on functional positioning of the mandible instead of artificially-manipulated and rigidly-fixed positioning by composite bite blocks would be desirable. However, the time requirement of current buccal scan techniques and the artificially applied pressure on the buccal soft tissues makes fixation inevitable at this stage. Smaller, more rapid devices or motion capture-capability may provide an opportunity to use more advanced clinical solutions in the future.

The most alarming result of our work was the EcD_ratio of 41.83 mm (SD = 14.89). This suggested that a small amount of translational component in the complex movement might cause enormous displacement of the calculated ICR. Mehl also described this phenomenon in his study [30]. The EcD_ratio of his published data would be 6:1, 9:1, and 18:1 for different amounts of opening (9.6°, 6.4°, 3.2°) [30]. The mean amount of opening in our transformations was 3.68° (SD = 0.78), but the mean of our EcD_ratio was still more than two times higher than Mehl’s 3.2° group’s 18:1 ratio. Mehl’s results suggested a negative correlation between the amount of opening and the EcD_ratio. The correlation value with our data was −0.659, which suggested similar tendencies. These differences in the results are not surprising due to the significant differences in the study designs. Mehl’s study was free of the transverse and rotational components of the bite alignment errors, or the angular deviations of the axes; however, our study relied on clinically gained input, thus these factors might have important effects on the results.

Our findings and the results of Mehl’s study suggest that we still have limited knowledge of the ICR concept. We cannot fully describe in detail how the translational motion component of the complex movement of the mandible affects the position change of the ICRs [30]. The main source of this translational component is the normal function of the TMJ according to the ICR concept; however, technological and clinical sources of error might also have severe effects on the results. The legitimacy of these concerns is confirmed by two independent studies with different aims and methodologies. Our mathematical registration methodology might be a sufficient experimental tool for digital *“in vitro-like”* studies based on 3D modeling methods to describe the mathematical background of the reported EcD_ratios.

## Supporting information

S1 Fig A-H Graphical representation of the axes. The first set of axes for all four participants in 2D, on the level of the midsagittal plane, as the axes penetrate the plane. A, C, E, and G show the axes calculated as the first nonexcluded scan of the Bite0A position was used as the target of rotation for all nonexcluded scans of all opened bites. The opened scan of most centrally positioned axis (“x/+” marked symbols on the figures) was used to show the effect of error on the closed bites, thus all nonexcluded closed scans were used as the target of that chosen opened scan: B, D, F, H.
(TIF)

S1 Table Results. The relevant variables describing the differences in the axes of the same bite groups for the demonstrated first sets of each participant as shown in S1A–S1H Fig.
(DOCX)

S2 Table Data underlying the findings in the manuscript presented on the request of Reviewer #1 during revision. Not cited in text.
(XLSX)

S1 Text Detailed description of the chairside protocol. (DOCX)

S2 Text Detailed description of the CLVs method. (DOCX)

S1 Video Tutorial video for the reproduction of the CLVs method. (MP4)
