# Supplementary figures and images for: A novel approach for determining instantaneous centers of rotation of the mandible with an intraoral scanner: A preliminary study
Source: PLoS One. 2023 May 3;18(5):e0285162. doi: 10.1371/journal.pone.0285162 (PMC10156001; doi:10.1371/journal.pone.0285162)

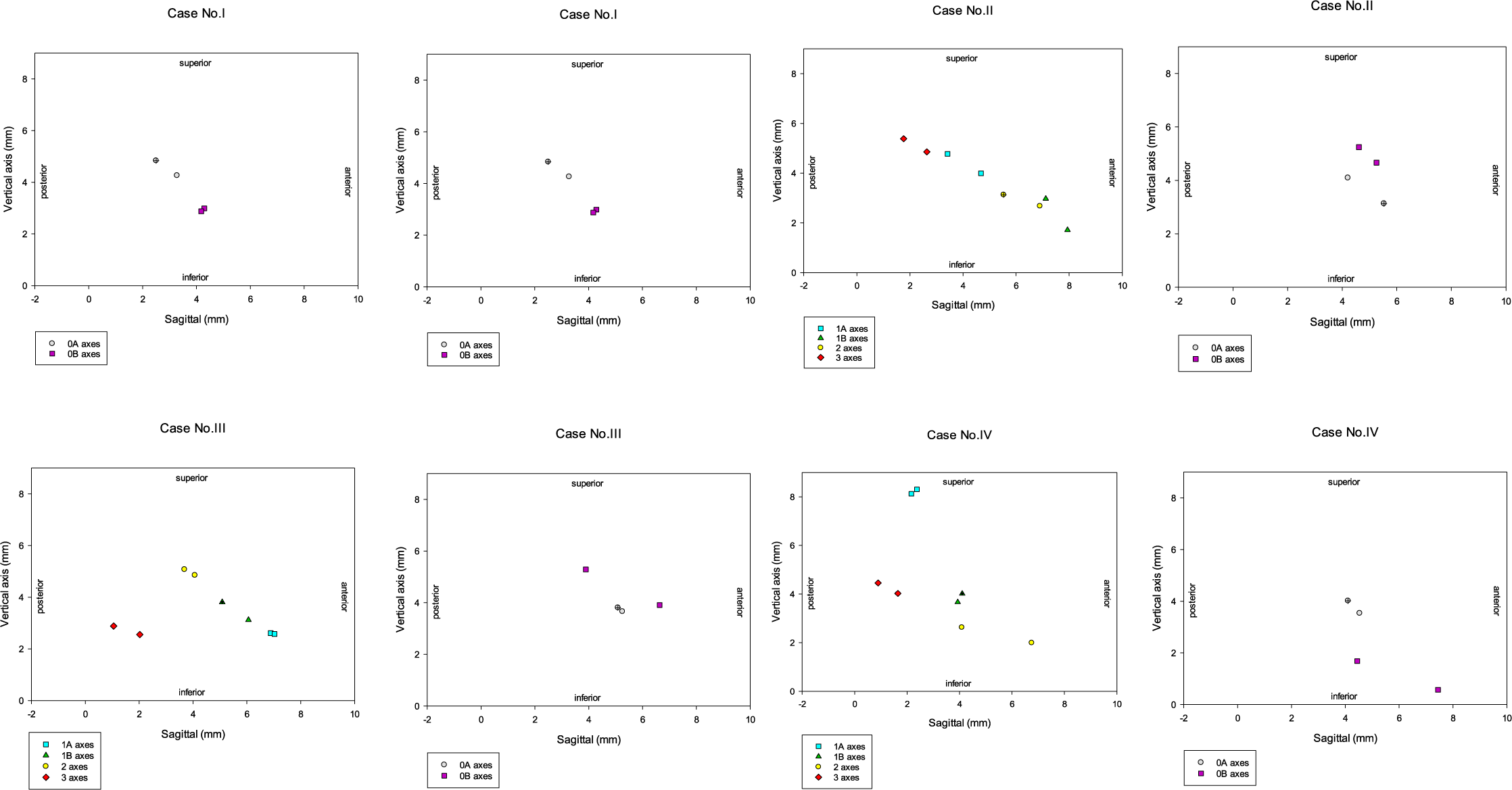

Supplement: S1 Fig — A-H Graphical representation of the axes. The first set of axes for all four participants in 2D, on the level of the midsagittal plane, as the axes penetrate the plane. A, C, E, and G show the axes calculated as the first nonexcluded scan of the Bite0A position was used as the target of rotation for all nonexcluded scans of all opened bites. The opened scan of most centrally positioned axis (“x/+” marked symbols on the figures) was used to show the effect of error on the closed bites, thus all nonexcluded closed scans were used as the target of that chosen opened scan: B, D, F, H. (TIF) [file pone.0285162.s001.tif]
