# Supplementary material for: A novel approach for determining instantaneous centers of rotation of the mandible with an intraoral scanner: A preliminary study
Source: PLoS One. 2023 May 3;18(5):e0285162. doi: 10.1371/journal.pone.0285162 (PMC10156001; doi:10.1371/journal.pone.0285162)
Supplement: S1 Table — The relevant variables describing the differences in the axes of the same bite groups for the demonstrated first sets of each participant as shown in S1A–S1H Fig. (DOCX) [file pone.0285162.s002.docx]

**S1 Table. Results.** The relevant variables describing the differences in the axes of the same bite groups for the demonstrated first sets of each participant as shown in S1 Fig A-H.

|  | **Bite No.** | **rms_error of the meshes** | **D_axis_** | **Angular deviation** | **Avarage degree of closure** |
| --- | --- | --- | --- | --- | --- |
| **Case No I.** | 1A | 0.02666 | 1.7301939 | 0.2764394 | 3.267433 |
|  | 1B | 0.04594 | 2.0054662 | 0.8547479 | 3.344128 |
|  | 2 | 0.02244 | 0.843289 | 0.1091274 | 4.275812 |
|  | 3 | 0.02403 | 1.1422391 | 0.4761498 | 4.991254 |
|  | 0A | 0.01946 | 1.0681589 | 0.3325534 | 4.336472 |
|  | 0B | 0.01724 | 0.3580295 | 0.1586396 | 4.453514 |
| **Case No II.** | 1A | 0.04213 | 1.5194053 | 0.1393362 | 3.138568 |
|  | 1B | 0.036 | 1.7084965 | 0.5935091 | 2.67404 |
|  | 2 | 0.04897 | 1.4811347 | 0.1916527 | 3.589277 |
|  | 3 | 0.04452 | 2.7054238 | 1.2527908 | 4.572857 |
|  | 0A | 0.03385 | 1.7744968 | 0.4208962 | 3.513305 |
|  | 0B | 0.02917 | 1.1642855 | 0.4315877 | 3.59428 |
| **Case No III.** | 1A | 0.0198 | 0.4817969 | 0.2443923 | 3.937324 |
|  | 1B | 0.0538 | 2.6061886 | 1.2490971 | 4.008067 |
|  | 2 | 0.033998 | 0.4914431 | 0.1442168 | 4.584192 |
|  | 3 | 0.03474 | 1.4402434 | 0.5868475 | 4.282165 |
|  | 0A | 0.02215 | 0.6135103 | 0.308787 | 4.003659 |
|  | 0B | 0.01463 | 0.9787009 | 0.4681132 | 3.956225 |
| **Case No IV.** | 1A | 0.0238 | 0.4550763 | 0.2330047 | 2.406446 |
|  | 1B | 0.03651 | 1.8031879 | 0.8386728 | 2.266881 |
|  | 2 | 0.08841 | 2.9733453 | 0.7690028 | 3.489292 |
|  | 3 | 0.02525 | 0.865001 | 0.0682702 | 3.556905 |
|  | 0A | 0.01707 | 0.8949235 | 0.3589645 | 2.272324 |
|  | 0B | 0.06262 | 3.2833862 | 0.8284672 | 2.313637 |
