# Supplementary material for: A novel approach for determining instantaneous centers of rotation of the mandible with an intraoral scanner: A preliminary study
Source: PLoS One. 2023 May 3;18(5):e0285162. doi: 10.1371/journal.pone.0285162 (PMC10156001; doi:10.1371/journal.pone.0285162)
Supplement: S1 Text — (DOCX) [file pone.0285162.s004.docx]

## Detailed description of the chairside protocol.

Six inter-occlusal registrations were made: two in closed and four in opened positions for each participant. The strategy of multiple inter-occlusal registrations was designed to control the clinical registration method. Each position was scanned three times continuously to control bite alignment accuracy separately from clinical procedures (Fig 1). Preliminary investigations showed that the alignment accuracy was highly unstable, which is consistent with the findings of Nilisson et al. [1]. We also found that scanning the same subject two times in the same position produced unsatisfactory results on occasion. On average, two of three bite scans were placed relatively close to each other, while one of the three scans was positioned relatively further away. We eventually decided to carry out three scans continuously without any movement, but only the best visually-selected two scans of three were used for subsequent processing. These consecutive buccal scans that were carried out in the same inter-occlusal record formed *a group*; for a more fluid formulation, we will henceforth refer to them using the colloquial term *"bite groups"*.

Due to the stationary position that had to be maintained for a long period of time, we used a new composite-based method instead of the traditional methods of inter-occlusal registration.

Four inter-occlusal records were made in an open stage during mandibular closure in an upright chair position. Plates of 1 mm, 2 mm, and 3 mm thickness were formed from dental composite materials and subsequently light cured before use. An uncured composite bundle was placed on one of the surfaces of the plates and pushed against the occlusal surface of the upper premolars. The composite plates were modified with drilling instruments until simultaneous contact was achieved on both sides during chin-point guidance that was applied with light force only. After repeated satisfactory patient feedback, a flowable composite material was applied in small quantities on the expected contact surface, and this was light cured after guided closure. The bite blocks were also tested in a horizontal position with bimanual manipulation as described by Dawson, and the scans were subsequently carried out in that position [2]. Two pairs of 1 mm plates and one pair of each 2 mm and 3 mm plates were used (These bite groups are referred to as Bite1A, Bite1B, Bite2, and Bite3; see Fig 1). Three buccal scan scenarios for each pair of bite blocks were carried out continuously without opening.

Inter-occlusal registrations at the first contact (Bite0A and Bite0B) were made in a horizontal position. A flowable composite material was applied on the palatal cusps of the upper first molars and premolars, and the material was then light cured after guided closure. Three consecutive buccal scan scenarios were carried out without opening, and then the composite remnants were cleared. The closed inter-occlusal registration procedure was repeated again.

If any error was detected during the buccal scan, it was repeated immediately (such a repeated scan was needed because of increased saliva flow that was depicted on the buccal scan image; that bite group consisted of four scans instead of the normal three, two of which were excluded later; see Fig 1).

**References**

1. Nilsson J, Richards RG, Thor A, Kamer L. Virtual bite registration using intraoral digital scanning, CT and CBCT: In vitro evaluation of a new method and its implication for orthognathic surgery. J Cranio-Maxillofacial Surg. 2016;44: 1194–1200. doi:10.1016/j.jcms.2016.06.013

2. Dawson PE. Evaluation, diagnosis, and treatment of occlusal problems. 2nd ed. St. Louis: Mosby-Year book; 1989.
