# Supplementary material for: A novel approach for determining instantaneous centers of rotation of the mandible with an intraoral scanner: A preliminary study
Source: PLoS One. 2023 May 3;18(5):e0285162. doi: 10.1371/journal.pone.0285162 (PMC10156001; doi:10.1371/journal.pone.0285162)
Supplement: S2 Text — (DOCX) [file pone.0285162.s005.docx]

## Detailed description of the CLVs method.

The basic idea behind our study is that, if upper scans of all 18 registrations are identical, furthermore they are positioned identically in the 3D space. Nevertheless, the lower *(also identical)* scans kept their unique positions as determined by the buccal scans, than the individual ICRs of the opened lower meshes (collated to the closed lower ones) could be calculated.

However, two obstacles prolonged the digital workflow:

- The 3shape software positioned the upper mashes randomly in digital space, but not in identical positions, which made another, time-consuming alignment process inevitable.
- The other unfortunate trait of the scanner, that although the in-built post process feature had been carried out before duplication, it is mandatory to repeat it after the buccal scans. This event recalculated and altered the previously prepared surfaces of the upper and lower scans as well, thus the duplicated meshes were no longer identical.

These circumstances prolongated the post-scan digital work significantly and made it difficult to conduct large scale studies. It reduced the verifiability of the scanner and obstructed monitoring of the alignment accuracy, which would have been desirable [1]. To eliminate these problems, we used an alignment process combined with a substitutional phase carried out in Blender 2.79.2 (Blender Foundation, Amsterdam), which is open-source general-purpose modeling program. This phase of the work contained the following four steps:

1. An upper scan was positioned at the origin of the 3D coordinate system with the X-axis parallel to the transverse direction, Y in the sagittal direction, and Z in the vertical direction. The occlusal plane was placed perpendicular to the horizontal plane and the palatal raphe to the sagittal plane. This mesh was used as a reference and all other upper scans were aligned to it in a total overlapping position.
2. Despite the fact that the second postprocess phase slightly altered the surfaces of the meshes, we found the edges and remote parts stayed relatively stable compared to the ones that were not postprocessed before duplication. Three characteristically located vertices (CLVs) were selected along the gingival edges of the reference mesh at distant positions in *Edit Mode*, and a *Face* was generated (Fig 2). This *Face* had a unique *Center* and a *Local Orientation*. The *Local Orientation* was saved. A designated object, an *Empty* was transferred to the *Center* of the *Face* and aligned to its previously saved *Orientation*. Then, the first of the 18 pairs of meshes were imported and the analog CLVs of the upper arch were selected and the previous sequence was carried out until the new *Empty* was placed and oriented correctly. The upper and lower meshes were bound to the second *Empty* with the *Parent Object* function, thus all translational movement and rotation of the *Empty* also affected the scans. The second *Empty* was transferred to the original *Empty’s* position and aligned to its *Orientation*, thus perfect position and orientation in space could be achieved. The perfect overlap of the upper mashes was visually verifiable as those pars shined in a bright orange color. If any part of the transferred object remained black, then one or more of the CLVs were affected by the postprocess, and the sequence was repeated with different CLVs. The steps were repeated until all upper and lower scans were in their designated position.
3. The exclusion of one of the three scans from the same bite group was carried out at this stage (if four scans were carried out on suspicion of clinical errors, then two meshes were excluded blindly; see above). In the majority of cases, exclusion was an obvious choice after a thorough visual inspection. Only one bite group of all bite groups for all participants was problematic. In that case, all three scans were poorly positioned and logical choices could not have been made. That bite group was rescanned the next day with satisfactory results (Fig 1).
4. At this stage, the lower scans were in their correct position; however, the meshes were not identical. A modified version of Step 2 was carried out to replace them to so they were identical. On the first lower mesh, new CLVs were picked and transformed to a *Face*, and by the previously described steps, an *Empty* was placed and oriented. This *Empty* was *Parented* to the lower mesh and then duplicated multiple times. One of the duplicates was positioned at the next lower scan by the CLVs and *Empty* method. This process was repeated until one of the duplicates was aligned at all the original lower scans. By the end of this substitution phase, the cloned scans were in a proper position and they were identical except for their unique position in 3D space.

**References**

1. Nilsson J, Richards RG, Thor A, Kamer L. Virtual bite registration using intraoral digital scanning, CT and CBCT: In vitro evaluation of a new method and its implication for orthognathic surgery. J Cranio-Maxillofacial Surg. 2016;44: 1194–1200. doi:10.1016/j.jcms.2016.06.013
